# Limits of Detection of Gravimetric Signals on Earth

**DOI:** 10.1038/s41598-018-33717-z

**Published:** 2018-10-17

**Authors:** S. Rosat, J. Hinderer

**Affiliations:** Institut de Physique du Globe de Strasbourg, UMR 7516, Université de Strasbourg/EOST, CNRS, Strasbourg, France

## Abstract

Gravimetry is a well-established tool to probe the deep Earth’s processes. Geophysical signals coming from the deep Earth, like the inner core free oscillations, have however never been detected. Challenging quests raise the question of the limits of detection of elusive signals at the Earth’s surface. Knowledge of the instrumental limits and of the environmental noise level at a site is fundamental to judge the true sensitivity of an instrument. We perform a noise level comparison of various gravimeters and a long-period seismometer at the J9 gravimetric observatory of Strasbourg (France) to provide a reference of instrumental performances. We then apply a three-channel correlation analysis of time-varying surface gravity from superconducting gravimeter records to isolate the instrumental self-noise from the environmental noise. The self-noise coherence analysis shows that the instrumental noise level remains flat towards lower frequencies till 10^−4^ Hz. At seismic frequencies, the self-noise is well explained by a Brownian thermal noise model. At daily and sub-daily time-scales, self-noise is increasing with the period but to a much lesser extent than observed noise level. Observed Earth’s ambient noise level at sub-seismic frequencies is hence mostly due to unmodeled geophysical processes. At hourly time-scales, our ability to detect elusive signals coming from the deep Earth’s interior is not limited by the instrument capability but is mostly due to the environmental effects.

## Introduction

Our understanding of the deep Earth’s interior has made great progress with seismology and geomagnetism. Resolving the Earth’s density within the core is however still struggling large uncertainties^1–4^. Theoretical studies have demonstrated that gravity changes from core flows could be detected in high-precision gravity measurements^5^. Gravity variations resulting from mass redistribution within the core were indeed detected at decadal to sub-decadal timescales from satellite gravity measurements using GRACE data^6^ and interpreted as dissolution-crystallization process at the core-mantle boundary^7^. A positive density anomaly for the lower-mantle Large Low-Shear-Velocity Provinces was recently proposed to corroborate a 6-year signal observed in surface GPS displacement and geomagnetic data, but the associated surface gravity effect of a few tens of nanogals (~10^−11^ g) was not detected neither by space nor ground gravity measurements^8^. Continuous ground measurements of the time-variable gravity currently suffer from a small instrumental drift and episodic small offsets of various origins (earthquakes, rainfalls, etc.) that make the detection of small decadal signals unreliable. At daily to sub-daily time-scales, the core undertones^9^ and the inner core free oscillations^10,11^ have never been detected^9,12^. Our knowledge of the density structure within the core and at the inner core boundary would greatly benefit from an observation of these normal modes. Space satellite gravity measurements have a precision at the microgal level (10^−11^ g) but a time resolution of 10 days or longer and a spatial resolution around 400 km^6^. For global dynamic processes at shorter time-scales, like the core and inner core modes, we still need ground gravity measurements. The challenging quests for sub-daily signals from the deep core raise the question of whether we may expect to be able to detect in the future such elusive signals at the Earth’s surface. Knowledge of the instrumental limits and of the environmental noise level at a site is fundamental to help responding to such a general question by discriminating the sensitivity of the sensor from the ambient conditions. Knowing which instruments should be privileged in the search for a specific process can be achieved only when we have some reference noise levels and detection thresholds. Inferences of noise levels at various worldwide sites have been done in seismology resulting in the widely used Low Noise Model (NLNM)^13^. Berger *et al*.^14^ have used stations of the Global Seismographic Network (GSN) to compute Power Spectral Densities (PSDs) in a standardized procedure resulting in an updated ambient Earth noise model. With the development of a global network of Superconducting Gravimeters (SGs)^15,16^ in the nineties, some studies compared the noise levels of SGs with spring gravimeters^17,18^ and with long-period seismometers^19,20^ demonstrating that SGs are the most adapted instruments for studying geophysical signals below 10^−3^ Hz^21–24^. Despite a noise level below the ambient Earth noise model, sub-seismic signals coming from the Earth’s deep interior, like the translational oscillation of the inner core or the free inner core nutation, have not been observed yet^12,25,26^.

The problem raised in the present paper is whether it is possible to achieve a lower noise level so that we have a chance to detect in the future signals coming from the deep Earth. In other words, are we limited by the instrumental capabilities or is it still possible to decrease the observed noise level by improving our modelling, in particular of the atmospheric and hydrological fluid layers that cover a wide range of temporal and spatial scales?

Sources of noise can be environmental, anthropogenic or instrumental. In order to respond to this problem, we need to be able to quantify which part of the noise stems from the instrument itself and which part is caused by ambient environmental or anthropogenic processes. It is, however, impossible to discriminate between them from records of one single instrument at any one site.

## Data and Methods

Between July 2016 and September 2017, the J9 gravimetric observatory of Strasbourg (France) hosted six Superconducting Gravimeters (SGs) manufactured by GWR. Besides the compact old type GWR SG C026 that has been recording nearly continuously since 1996, five SGs of the latest generation have been installed: an iOSG-type (#23) recording since February 2016 and four iGrav-type SGs (#15, #29, #30 and #31) recording temporarily between July 2016 and September 2017. Besides the SGs, the J9 site periodically hosted several other gravimeters and a long-period STS-2 seismometer. These are: (i) LaCoste-Romberg Earth Tide gravimeter ET#11, (ii) Micro-g LaCoste gPhone #54, (iii) Scintrex CG5 (#40691), (iv) a STS-2 seismometer and (v) the FG5 #206 absolute gravimeter. While some data from the SG C026 gravimeter, spring and free-fall gravimeters and the seismometer have already been published elsewhere^18^, here, we make use of the additional data set from the iOSG #23 and the four iGravs (#15, #29, #30 and #31) as well as from a second CG5 gravimeter (#9379). Please note that the time-series from the C026, gPhone #54 and CG5 #40691 acquired in 2016 and 2017, and considered here, are different to the time-series published earlier^18,27^. For the FG5 absolute gravimeter, we used drop measurements (every drop corresponds to the free fall of the object) which is presently performed every 10 s. In Rosat *et al*.^18^, the PSD for the FG5 was computed on set values with hourly sampling. Table 1 summarizes the available time-series for each instrument. Comparing the time-varying gravity records from the SGs gives us the unique opportunity to extract the coherent noise by cross-correlation analysis of the time-series and hence to distinguish the instrumental self-noise from the environmental noise existing at the J9 site.

**Table 1:** Start and end times of available time-series used in this paper for each instrument recording at the J9 gravimetric observatory of Strasbourg (France).

Instrument	Start time	End time	Remarks
SG C026	1996-07-17	Still recording	
iOSG #23	2016-02-06	Still recording	
iGrav #15	2017-06-24	2017-10-19	
iGrav #29	2016-07-28	Still recording	
iGrav #30	2016-07-16	2017-06-18	
iGrav #31	2016-07-16	2017-07-17	
L&R ET#11	2011-07-01	2014-06-09	
CG5 #9379	2017-01-13	2017-06-27	7 time segments within the interval
CG5 #40691	2016-12-06	2017-06-27	9 time segments within the interval
gPhone #54	2016-04-01	2016-12-12	
FG5 #206	2017-07-03	2017-07-11	
STS-2	2011-07-01	2014-09-13	

### Noise levels estimates

**Figure 1:**
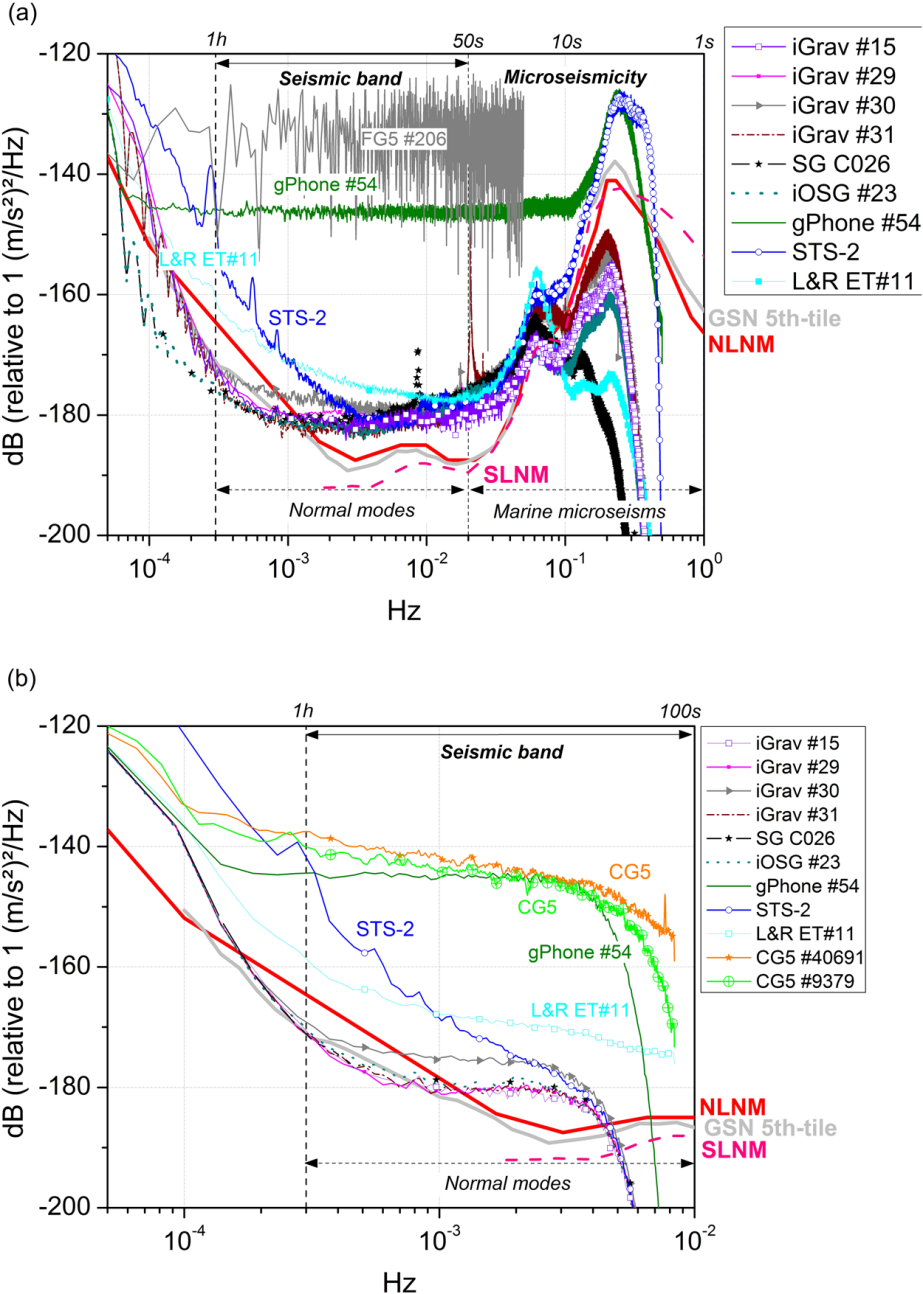
Fifth percentile of PSD noise levels computed on (**a**) 1-second; (**b**) 1-minute, sampling data of the six GWR Superconducting Gravimeters (C026, iOSG #23, iGrav #15, iGrav #29, iGrav #30 and iGrav #31), of the STS-2 seismometer, of the Micro-g LaCoste gPhone #54 and of the LaCoste-Romberg ET#11 gravimeter that were recording at the J9 Gravimetric Observatory of Strasbourg (France). The FG5 (#206) drop files were also used to compute the corresponding PSD. The New Low Noise Model (NLNM) is represented by the thick red line and the SLNM is represented by the thick dashed pink line. In dashed gray lines, we have plotted the 5th percentile of the PSD levels for the Global Seismographic Network (GSN 5th-tile).

Similarly to the procedure for GSN stations^14^, we have computed power spectral densities (PSDs) of calibrated data using a modified Welch periodogram^28^ method applied on 12 h time windows overlapped by 6 h. From the density distribution of PSDs, we have computed the 1st, 5th, 25th and 50th percentiles but we have selected only the 5th-tile for the plots in Fig. 1 to be compared with the GSN noise models^14^. The NLNM model^13^ and the more recent SLNM^29^ are also plotted for reference. Note that the NLNM corresponds to the lower envelope of seismic PSDs computed at that time, so it represents the lowest noise level reached by seismometers. In Fig. 1a the PSD noise levels were computed on raw 1-second time-series while in Fig. 1b, 1-second data were low-pass filtered and decimated to 1 minute.

### Three-channel analysis of SG records

**Figure 2:**
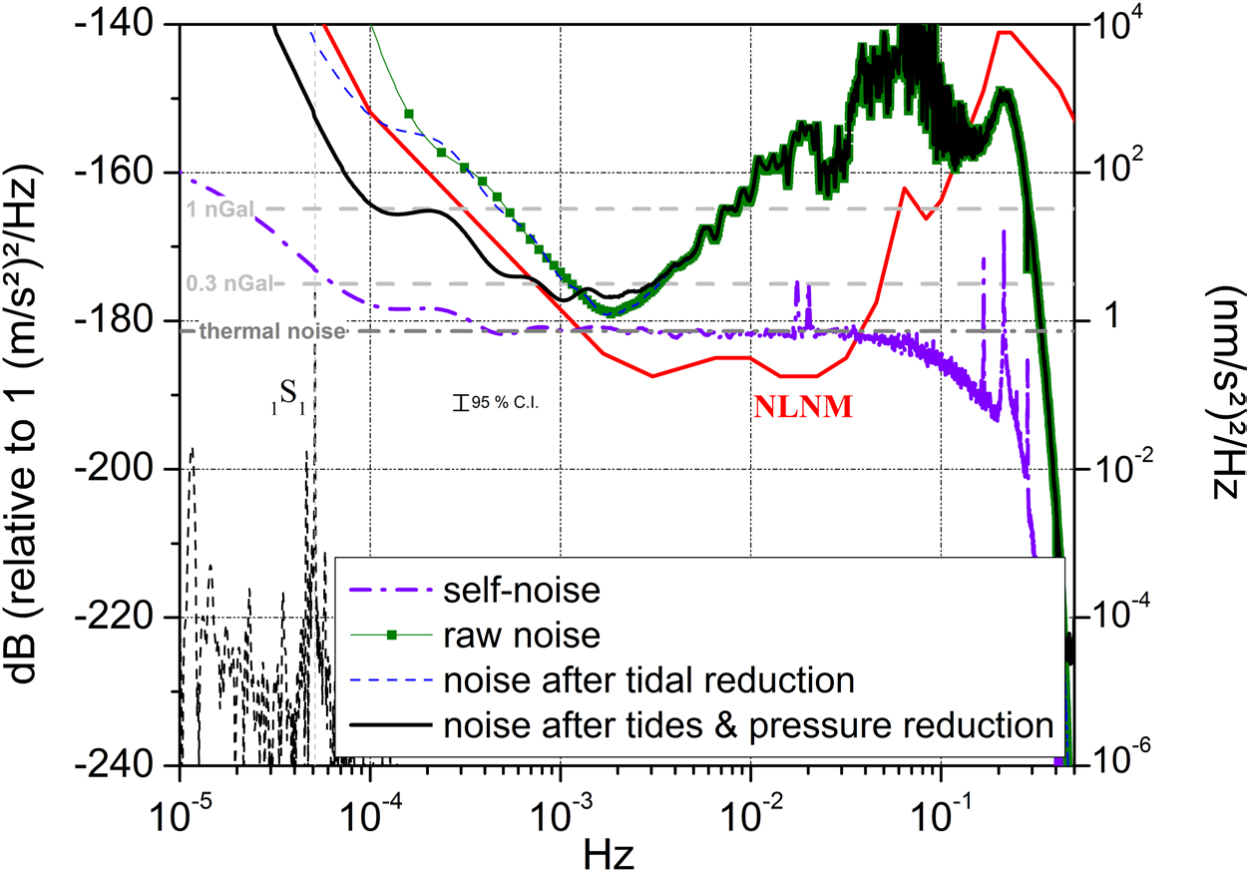
Results for iGrav #29 of the three-channel correlation analysis applied on the 1-second data on a 15 day time period (2017, August 10th to 25th) between iGrav #29, iGrav #15 and iOSG #23. Observed noise level (“raw noise”) and remaining noise levels (5th percentile) after subtraction of a local tidal model and after removing tides and the local atmospheric pressure effect are respectively plotted as green squares, blue dashed and black lines for iGrav #29. The extracted self-noise is plotted as magenta dashed line. The thermal noise model for iGrav #29 is indicated as a horizontal dashed and dotted gray line. The low noise model is plotted in red. Horizontal dashed gray segments represent the levels of detection of harmonic signals of respective amplitudes 0.3 and 1 nGal. The dashed black line is the predicted PSD amplitude for the Slichter mode (_1_S_1_) excited by the surface atmospheric ECMWF pressure field. The 95% confidence interval (C.I.) of the PSD estimate is indicated.

We applied a three-channel correlation technique^30^. For that we computed the PSDs and the cross-PSDs of the various calibrated SG records using a modified Welch periodogram method applied by averaging 9 segments of 48-h SG time-windows overlapped by 75% on two selected time-periods of 15 days. Several time-periods, May 23–June 2nd, 2017 and August 10–25, 2017 were chosen because of the availability of at least three instruments without any human intervention. The resulting observed and self-noise PSDs are plotted in Fig. 2 for the iGrav #29. The self-noise PSD was obtained by subtracting the common coherent noise between iGrav #29, iGrav #15 and iOSG #23. Only results for data from iGrav #29 recorded in August 2017 are shown here but other analyses are displayed as auxiliary online material. Observed noise levels computed from raw data are also plotted and compared with PSD noise levels after subtraction of the solid and ocean tides using a local tidal model and of the local atmospheric pressure effect using a barometric nominal admittance of −3 nm/s^2^/hPa. The observed response of SGs and iGravs to atmospheric pressure changes at J9 site gives an admittance value of −2.8 nm/s^2^/hPa. Local atmospheric pressure reduction is known to be efficient at frequencies lower than 2 mHz^31^. Using a nominal admittance of −3 nm/s^2^/hPa instead of −2.8 nm/s^2^/hPa makes the PSD level higher by 0.5 dB at 10^−4^ Hz which is within the 95% confidence interval of the PSD estimate.

### Thermal noise model

Theoretically, the noise of the SG sensor is due to the thermal noise associated with Brownian motion in a simple damped mechanical oscillator^32,33^. The expression of the power spectral density of a damped harmonic oscillator due to Brownian motion can be written:

1 $${P}_{thermal}=4{k}_{B}T\frac{{\omega }_{0}}{mQ},$$

where ω_0_ is the natural frequency of the oscillator depending on the levitating current, *Q* its quality factor and *m* is the mass of the oscillating sphere; *k*_*B*_ is the Boltzmann constant and *T* the temperature within the sensor. The iOSG #23 has a heavier levitating sphere with *m* = 17.7 g while standard iGravs have a sphere weighting around 4 g, so that the thermal noise is lower for iOSG #23 if the eigenfrequency (and spring constant) or quality factor does not compensate the mass change (cf. Eq. (1)). We used the harmonic oscillator parameters evaluated by GWR for iGrav #29 and iOSG #23 (Warburton, personal communication) and summarized in Table 2.

**Table 2:** Harmonic oscillator parameter values used to compute the spectral acceleration-noise power density of the thermal noise due to Brownian motion.

Parameter	Unit	iGrav #29	iOSG #23
Mass *m*	g	4.02	17.67
Frequency *f*_0_	Hz	0.24	0.10
*Q*		0.142	0.05
Spring constant *k*	N/m	0.0090	0.0076
Damping factor *b*	kg/s	0.051	0.232
Power Spectral Density	dB	−181	−188

## Results

At seismic frequencies lower than 3 * 10^−3^ Hz, SGs present the lowest noise magnitudes and the other spring gravimeters (gPhone #54 and CG5) have the highest noise levels, while the ET#11 gravimeter and STS-2 seismometer lie in-between. At higher frequencies, the STS-2 has similar performances than the SGs. At sub-seismic frequencies, the SGs still have the best performances while the ET#11 spring gravimeter and the other spring gravimeters reach similar noise magnitudes. We refer to the work by Rosat *et al*.^18^ for detailed comparisons and self-noise analysis for these instruments. The sampling rate of the two CG5 being of one minute in this study, the PSD levels for these spring gravimeters are plotted only in Fig. 1b. As for the FG5 noise level, using 10 sec drop values, we have slightly reduced the PSD level which was around −125 dB in a previous study^18^ to −130 dB at 10^−4^ Hz. It is well-known that absolute measurements are aliased by the microseismic noise^34^ explaining the rather flat FG5 noise spectrum.

We can see on Fig. 2 that at seismic frequencies larger than 10^−3^ Hz and before the attenuation due to the low-pass anti-aliasing filter, the thermal noise model perfectly agrees with the extracted self-noise PSD for iGrav #29. Same results are obtained for iGrav #15 (See Fig. S1). Because of the heavier levitating sphere, the thermal noise for iOSG #23 is below the NLNM seismic noise and a few decibels lower than the thermal noise of iGrav instruments. The self-noise of iOSG #23 is however about 5 dB larger than the thermal noise model in the seismic band (cf. Fig. S2). We suspect that the self-noise extraction technique is less efficient for iOSG #23 because of differences in the transfer function of this instrument with respect to iGrav instruments. Self-noise PSD for iGrav #30 is much higher because of the installation set-up (see Fig. S3). As a consequence, self-noise extraction of iGrav #31 based on data from the iGrav #29 and iGrav #30 is contaminated by the noise of iGrav #30.

Two parasitic noise peaks around 2 * 10^−2^ Hz are visible in the self-noise PSDs of Fig. 2. They are usually hidden by the micro-seismic noise and can only be visible when removing such an environmental noise. These vibrations have not been identified yet. They appear only in the gravity records and not in the other recorded auxiliary channels, like the tilt compensation records. So they do not seem to be due to ground motion. Please note that because of the three-channel correlation analysis, such a peak in the data of one instrument will be transmitted to other self-noise PSDs. Peaks around 0.2 Hz are due to the resonance modes of the levitating spheres of the sensors, for instance 0.24 Hz for iGrav #29 (see Table 2).

At sub-seismic frequencies lower than 10^−3^ Hz, the self-noise increases with period but to a much lesser extent than environmental noise. This is the first time we show that instrumental self-noise is not the major contributor to the observed increase of noise level towards lower frequencies. At longer periods, the instrumental drift of the SG will limit the capability of detecting decadal signals from the core.

Assuming white noise, the PSD level is a constant equal to σ^2^T_0_, where σ is the standard deviation of noise and T_0_ the sampling interval. For a pure undamped harmonic signal, the PSD is obtained by the relation PSD = A^2^NT_0_/4, where A corresponds to the amplitude of the sinusoid and N is the number of samples. Thus a harmonic signal becomes observable in white noise PSD when A^2^NT_0_/4 > σ^2^ T_0_, i.e. when A > 2σ/√N. Predicted levels of detection of harmonic signals of respective amplitudes 0.3 and 1 nGal (10^−11^ m s^−2^) are indicated in Fig. 2 to provide limits of detection of elusive signals.

As an example of a yet undetected geophysical signal, we mention the free translational oscillation of the inner core inside the fluid outer core. This normal mode of the Earth, noted _1_S_1_, has a predicted period of 5.42 h^35^ for a PREM-like Earth model^36^ and is often called Slichter mode or Slichter triplet^10^. We refer to previous works for theoretical computation of this mode^11,12,37–40^. The predicted PSD amplitude for the Slichter mode being continuously excited by the surface atmospheric ECMWF pressure field^12^ is also plotted on Fig. 2. We can see that the self-noise of iGrav instruments are close to the level of detection of the Slichter modes with a detection level reaching 0.3 nGal. A stacking method on S time-records, would improve the signal to noise amplitude by √S. The observed detection level at the _1_S_1_ frequency being close to 3 nGal, stacking 100 time-series would make the detection possible. Stacking 10 SG time-records with the noise level observed in Fig. 2 would enable to detect a 1 nGal signal at few hour time-scales.

At seismic frequencies, the resolution is even smaller, at the level of 0.1 nGal. The first detection of the seismic _2_S_1_ mode, first harmonic of the Slichter mode with amplitude of a few nGals, was indeed performed by stacking five SG records after the 2001 M_w_8.4 Peru earthquake^41^. We can see however that the obtained self-noise PSDs are still above the background noise as modelled by the NLNM in the 0.002 to 0.03 Hz frequency range. The potential detection of sub-nanogal transient gravity signal is limited by the background seismic noise and makes it challenging, but possible, to use SGs to detect gravito-elastic perturbations induced by a seismic rupture before the arrival of seismic waves^42,43^.

## Discussion

**Figure 3:**
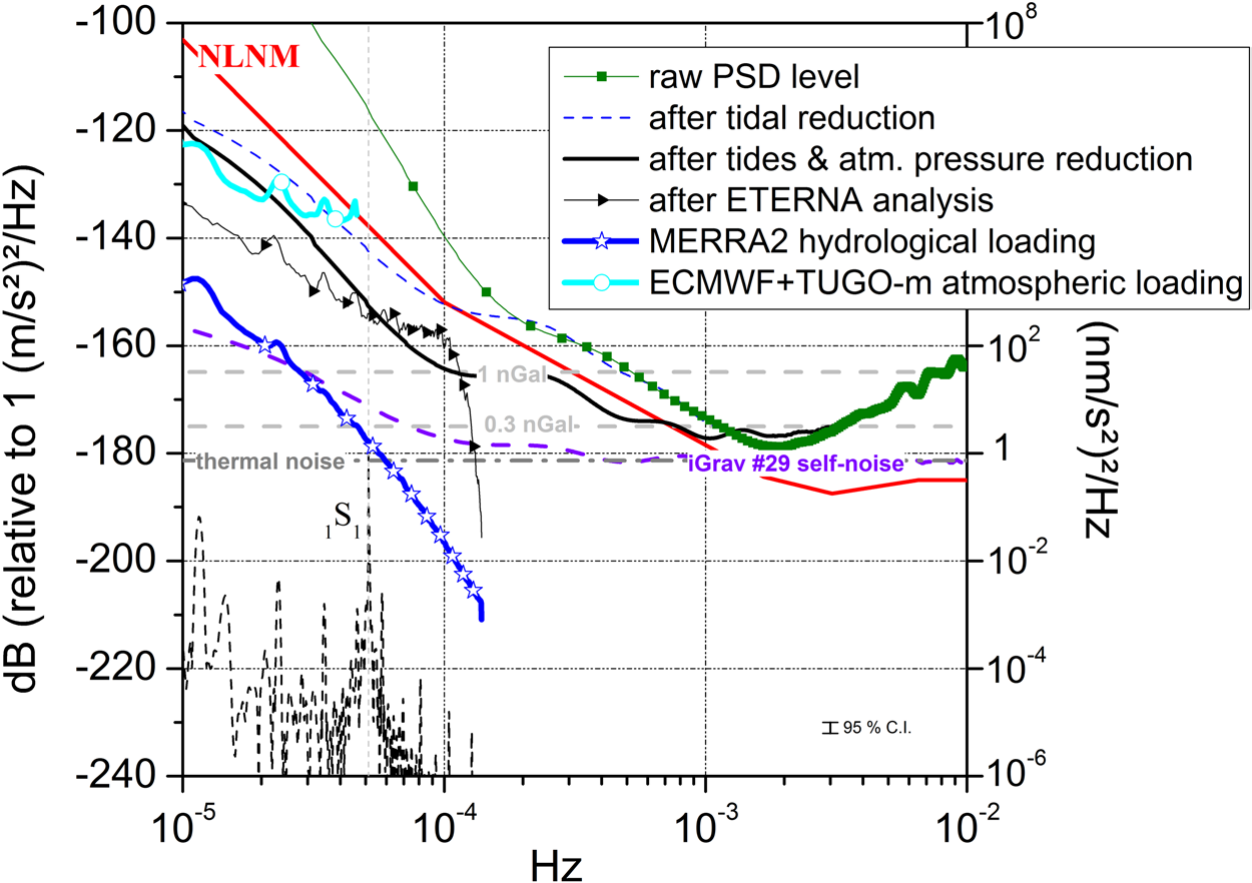
Sub-seismic noise levels for the iGrav #29 before and after tidal and atmospheric pressure reductions on a 15-day time period between August 10th and 25th 2017. Median noise levels computed for atmospheric (ECMWF with TUGO-m dynamic ocean response) and hydrological (MERRA2 model) loading at J9 are also plotted together with the iGrav #29 self-noise PSD and the NLNM. Predicted PSD amplitude for the Slichter mode (_1_S_1_) excited by the surface atmospheric ECMWF pressure field is plotted in dashed black line. The thermal noise model as well as the detection thresholds at 0.3 and 1 nGal is plotted in gray. The 95% confidence interval (C.I.) of the PSD estimate is indicated.

We show that self-noise PSDs of the iGravs at sub-seismic frequencies are at the level of detection of the Slichter mode and hence what limits our capabilities to detect this inner core signal is not instrumental. The slight increase of self-noise towards longer periods is quite overwhelmed by geophysical signals. Differences of noise levels between the self-noises and the observed PSD levels after removing tides and atmospheric pressure effects are hence due to geophysical processes that have not been reduced from gravimetric records. Using least-squares adjustment software like ETERNA^44^ better reduces the observed noise level than subtracting a local tidal model (Fig. 3) but the PSD is still 20 dB larger than the self-noise.

We computed PSDs for the available atmospheric and hydrological surface loading products using a modified Welch periodogram computed on 15-day segments overlapped with 75% and the median was taken. As shown in Fig. 3, the largest energy at sub-seismic frequencies comes from the atmosphere. Present atmospheric loading computation was performed using ECMWF pressure field products^45,46^. The oceanic response to atmospheric forcing is modelled using the barotropic non-linear model based on 2-dimension gravity waves model, called TUGO-m 2D (Toulouse Unstructured Grid Ocean model 2D, ex-MOG2D)^47,48^. The spatial and temporal resolutions are respectively 0.25° and 3 hours. At hourly time-scales, using this loading computation in addition to the local air pressure effect calculated using a barometric admittance does not further improve the reduction of noise.

However correcting for the dynamics of atmospheric mass changes at such time-scales would need further improvements with respect to the classical loading contributions based on surface pressure and standard decrease of pressure with height; one should among others take into account the vertical convection of mass flows^49^.

In addition to the atmospheric masses, hydrological effects also have some impact at such frequencies to a much lesser extent (roughly 20 dB below). On Fig. 3 we have plotted the median PSD level obtained from the gravity effect computed with the MERRA-2^50^ hydrological loading model. The MERRA-2 atmospheric reanalysis product provides global estimates of land surface conditions using observation-based precipitation to force the land^51^. The spatial and temporal resolutions of MERRA-2 are respectively 0.625° (~50 km) in latitude and longitude and 1 hour.

Present hydrological and atmospheric modeling is clearly not relevant at hourly scales because of the difficulty to correctly model the dynamics of these fluid layers and the induced gravity effects. Only continuous monitoring of these surficial flows would help to correctly remove these environmental effects from gravity data in order to be able to detect elusive signals of the deep Earth.

## Conclusions

We have compared performances of various kinds of gravimeters in terms of noise levels. We have shown that SGs perform better than all other gravimeters and even better than long-period seismometer in the seismic band. We have then applied a three-channel correlation technique to five SGs of the latest generation. It is the first time that we are able to distinguish the environmental noise of a site from the purely instrumental noise of SGs. We have shown that the increasing noise with periods increasing from 1000 s to 6 h is predominantly geophysical and instrumental only to a minor degree. At seismic frequencies, the Brownian noise model of a damped oscillator agrees with the extracted self-noise. A further study is now needed to find the parts of the atmosphere and hydrology that contribute to the increase of noise level towards lower frequencies. Precise monitoring and modeling of these surficial mass flows are needed to further reduce the observed environmental noise in order to detect deep Earth signals. Innovative instruments such as superconducting gravity gradiometers^52^ or atom interferometers^53,54^ are currently being developed in the context of low-frequency gravitational-wave detectors. Superconducting Gravimeters have the potential to complement such innovative technologies in the challenging detection of elusive gravitational signals.

## Electronic supplementary material

Supplementary Information

## Data Availability

Data from the Superconducting Gravimeter C026 are available at http://cdg.u-strasbg.fr/PortailEOST/Gravi/gravimetrie-data.html. Other datasets are available upon request.
