# Supplementary material for: Limits of Detection of Gravimetric Signals on Earth
Source: Sci Rep. 2018 Oct 17;8:15324. doi: 10.1038/s41598-018-33717-z (PMC6193024; doi:10.1038/s41598-018-33717-z)
Supplement: Supplementary file 1 — Supplementary Information [file 41598_2018_33717_MOESM1_ESM.pdf]

**Limits of Detection of Gravimetric Signals on Earth**

**S. Rosat* and J. Hinderer**

## Auxiliary online material

In the main paper, we only show the results for the iGrav #29 on the time-period August 10 to 25, 2017. Self-noise analysis results for other instruments and May 23 to June 2nd, 2017 are displayed here. The three-channel correlation technique was applied by computing the PSDs and the cross-PSDs of the various calibrated SG records using a modified Welch periodogram method.

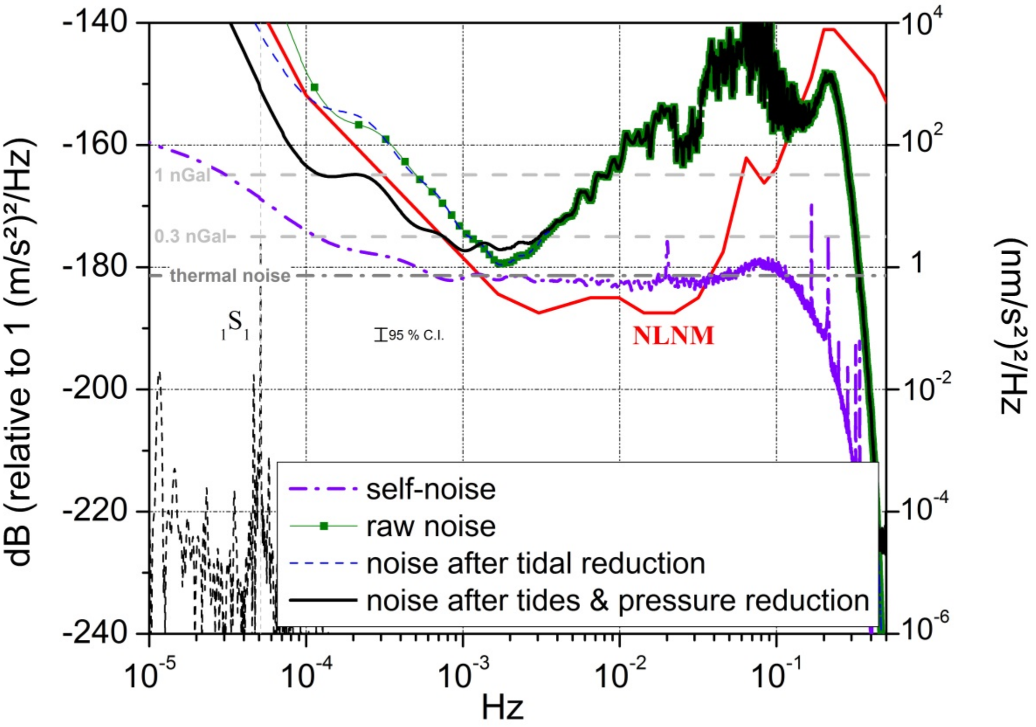


**Figure S1.** Results for iGrav #15 of the three-channel correlation analysis applied on the 1-second data on a 15 day time period (2017, August 10 to 25) between iGrav #29, iGrav #15 and iOSG #23. Observed noise level ("raw noise") and remaining noise levels (5th percentile) after subtraction of a local tidal model and after removing tides and the local atmospheric pressure effect are respectively plotted as green squares, blue open squares and black lines for iGrav #15. The extracted self-noise is plotted as magenta dashed line. The thermal noise model for iGrav #29 (not for iGrav #15 since we do no tknow the resonance parameters) is indicated as a horizontal dashed and dotted gray line. The low noise model NLNM is plotted in red. Horizontal dashed gray segments represent the levels of detection of harmonic signals of respective amplitudes 0.3 and 1 nGal. The dashed black line is the predicted PSD amplitude for the Slichter mode ($_1S_1$) excited by the surface atmospheric ECMWF pressure field. The 95 % confidence interval (C.I.) of the PSD estimate is indicated.

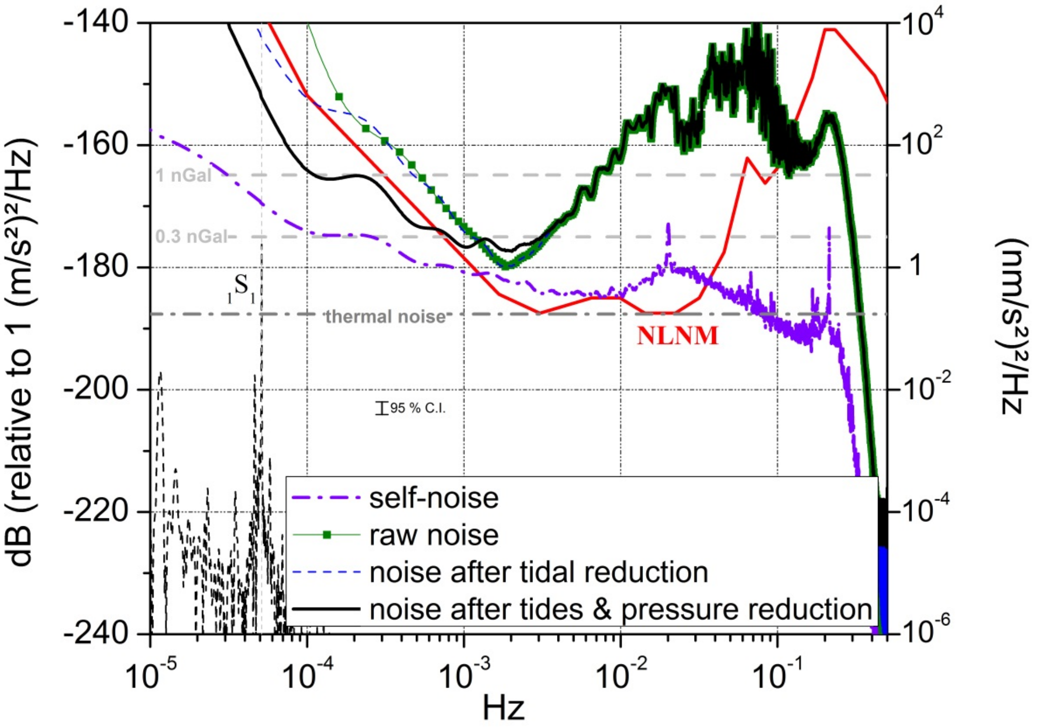


**Figure S2.** Results for iOSG #23 of the three-channel correlation analysis applied on the 1-second data on a 15 day time period (2017, August 10 to 25) between iGrav #29, iGrav #15 and iOSG #23. Observed noise level ("raw noise") and remaining noise levels (5th percentile) after subtraction of a local tidal model and after removing tides and the local atmospheric pressure effect are respectively plotted as green squares, blue open squares and black lines for iGrav #23. The extracted self-noise is plotted as magenta dashed line. The thermal noise model for iGrav #23 is indicated as a horizontal dashed and dotted gray line. The low noise model NLNM is plotted in red. Horizontal dashed gray segments represent the levels of detection of harmonic signals of respective amplitudes 0.3 and 1 nGal. The dashed black line is the predicted PSD amplitude for the Slichter mode ($_1S_1$) excited by the surface atmospheric ECMWF pressure field. The 95 % confidence interval (C.I.) of the PSD estimate is indicated.

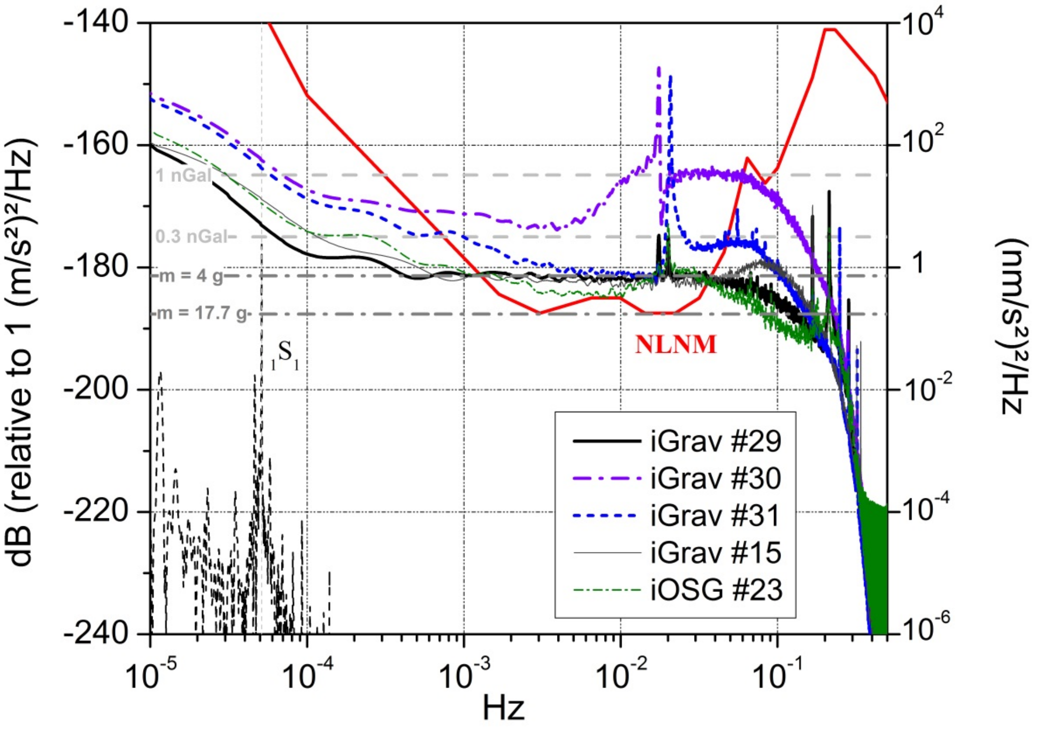


**Figure S3.** Results for iOSG #23, iGrav #15, iGrav #29, iGrav #30 and iGrav #31 of the three-channel correlation analysis applied on the 1-second data on a 15 day time period (May 23 to June 2$^{nd}$ or August 10 to 25, 2017) The thermal noise models for iGrav #23 and iGrav #29 are indicated as a horizontal dashed and dotted gray line respectively with the labels m = 17.7 g and m = 4 g. The low noise model NLNM is plotted in red. Horizontal dashed gray segments represent the levels of detection of harmonic signals of respective amplitudes 0.3 and 1 nGal. The dashed black line is the predicted PSD amplitude for the Slichter mode ($_1S_1$) excited by the surface atmospheric ECMWF pressure field.
